# The Genetic Diversity and Interspecific Transmission of Circovirus in *Rhizomys sinensis* in Guangdong, Southern China

**DOI:** 10.1155/2023/6668569

**Published:** 2023-11-09

**Authors:** Zhaowen Ren, Zi-Guo Yuan, Shengjun Luo, Chenglong Sun, Pian Zhang, Jieshi Yu, Xiaofan Chen, Jinping Chen, Yan Hua, Gang Wang, Hua Xiang, Rujian Cai, Jing Chen, Yuan Huang, Hao Yuan, Na Li, Xiaohu Wang, Ming Liao

**Affiliations:** ^1^Key Laboratory of Livestock Disease Prevention of Guangdong Province, Scientific Observation and Key Laboratory for Prevention and Control of Avian Influenza and Other Major Poultry Diseases, Ministry of Agriculture and Rural Affairs, Institute of Animal Health, Guangdong Academy of Agricultural Sciences, Baishigang, Wushan Street, Tianhe District, Guangzhou 510640, China; ^2^Guangdong Provincial Key Laboratory of Zoonosis Prevention and Control, College of Veterinary Medicine, South China Agricultural University, No. 483 Wushan Road, Tianhe District, Guangzhou 510642, China; ^3^Department of Molecular Biology, University of Texas Southwestern Medical Center, 5323 Harry Hines Boulevard, Dallas, TX 75390-9148, USA; ^4^Department of Microbial Resources Research, Agro-Biological Gene Research Center, Guangdong Academy of Agricultural Sciences, Guangzhou, China; ^5^Institute of Zoology, Guangdong Academy of Sciences, No. 105 Xingang West Road, Haizhu District, Guangzhou 510260, China; ^6^Guangdong Provincial Wildlife Rescue Center, No. 139 Yuxi Road, Tianhe District, Guangzhou 510520, China

## Abstract

Circoviruses are a group of small circular, single-stranded DNA viruses that belong to the family *Circoviridae*. They are known to infect a wide variety of animals. *Rhizomys sinensis* is a species of rodent that is the reservoir of many zoonotic pathogens. Our previous study identified many sequencing reads mapped to the genome of viruses in *Circoviridae* in *R. sinensis*. However, little is known about the circulation and genetic characterization of circoviruses in *R. sinensis*. This study identified three different circoviruses in samples from 195 *R. sinensis*. First, the bamboo rat circovirus is widely prevalent in *R. sinensis* in Guangdong Province, and all strains could be divided into three clades based on nucleotide substitutions at specific sites. Second, and more important, porcine circovirus 2 (PCV2) was isolated for the first time from *R. sinensis*, which expanded the host range of PCV2 and indicated extra procedures would be required to protect livestock from this virus. Finally, a novel circovirus phylogenetically close to the dromedary stool-associated circular ssDNA virus was detected in 86 (44.1%) samples, which may represent a new circovirus species. These results not only expand our understanding of the circovirus diversity in rodents, particularly in *R. sinensis*, but also underscore the importance of continued surveillance of viruses in wildlife populations, particularly in rodents, to prevent and control the spread of zoonotic pathogens.

## 1. Introduction

Circoviruses are the smallest known viruses with a small, circular, single-stranded DNA (ssDNA) genome [1]. The family *Circoviridae* was established in the mid-1990s [2]. Its members have ambisense genomes of ∼1.7–2.1 kb in length, containing two major open reading frames (ORFs). The replication-associated (Rep) gene lies in the viral sense strand, and the capsid (Cap) gene lies in the complementary sense strand, encoding the Rep and Cap proteins, respectively [2]. Recently, some new circular ssDNA viruses with genome sizes of more than 2.1 kb were also classified into the family *Circoviridae* [3–5]. Due to the high diversity of animal viruses with circular ssDNA genomes, members of the family *Circoviridae* have been further classified into two genera, *Circovirus* and *Cyclovirus* [2].

In the past decade, benefiting from the development of high-throughput sequencing technologies and metagenomes, many circoviruses have been identified in multiple new hosts [6], ranging from mammals (bats, dogs, minks, rodents, etc.) [7–10], birds [1], fishes [6], to even insects [11]. Due to being associated with several zoonotic diseases in vertebrates [12, 13], circoviruses have emerged as an economic concern over farmed livestock and poultry and a health concern over wildlife and humans [14].

Rodents (order Rodentia) are the most diverse and widest distributed mammals, with 33 families and 2,277 species (∼43% of all mammal species) [10]. They are a natural reservoir of many zoonotic viruses and intermediate reservoirs that serve as a bond between humans, domestic animals, arthropod vectors (ticks, mites, fleas), and other wildlife and cause the transmission of viruses [15]. Therefore, virus identification in rodent populations is of great value for public health. Over 200 rodent species from 12 families have been found in China [16]. Viral metagenome suggests that rodents can be infected by or carry numerous vertebrate-associated viruses, many of which can cause severe human diseases [10, 17–19]. Most virome studies have been focused on rodent species such as *Mus musculus*, *Rattus norvegicus*, *Rhombomys opimus*, and *Urocitellus undulates*. However, there is still a shortage of studies investigating virome associated with the *Rhizomys sinensis* species widely distributed in southern China. Recently, virome analysis by He et al. [20] identified seven vertebrate-associated RNA viruses in *Rhizomys pruinosus* (a species of bamboo rat). In one of our studies, more abundant vertebrate-related viruses covering 22 viral families were found in *R. sinensis* from Guangdong Province [21].

Interestingly, among many identified DNA viruses from *R. sinensis*, *Circoviridae* dominated more than 75% of the total viral reads. To further investigate circoviruses' circulation and genetic characterization in *R. sinensis*, 195 animals across Guangdong Province in China were sampled by throat and anal swabbing. Based on previous virome data, we redescribed viral composition in *R. sinensis* from Guangdong, focusing on the family *Circoviridae*. Various circoviruses were identified in bamboo rat populations, among which the bamboo rat circovirus (rodent-associated circovirus 7) was found widely throughout this region. Notably, the PCV2d genotype was identified for the first time in healthy *R. sinensis*, with the same antibody recognition regions, immunodominant decoy epitope, and a heparin sulfate-binding motif as observed in PCV2d of porcine origin. All findings in this study would significantly expand our understanding of virome characterization, especially the genetic diversity of circoviruses in *R. sinensis*.

## 2. Materials and Methods

### 2.1. Sample Collection

**Figure 1:**
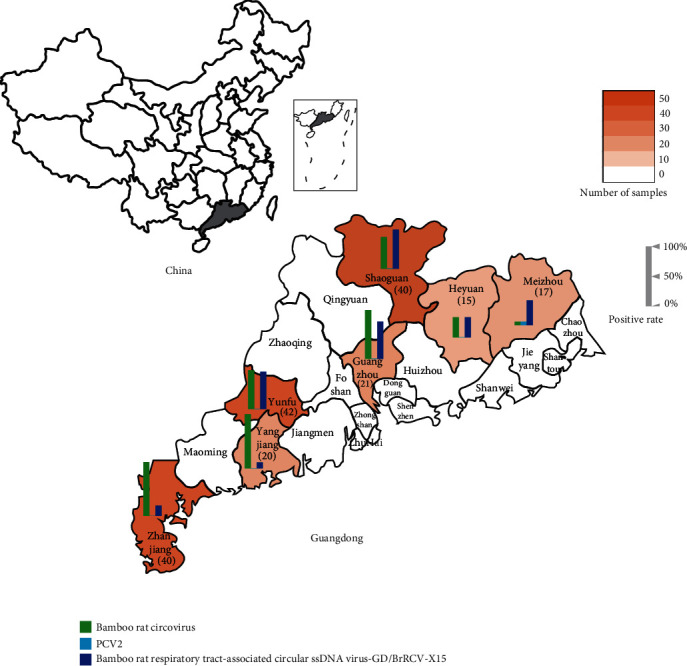
Summary of the samples and circoviruses identified in this study. The gray area is the geographical location of Guangdong Province (upper left corner) in China. Sample size from each city was indicated with color depth on the map and listed in parentheses under the city's name. The height of the bar graphs represented the positive rate of detected circoviruses in each city.

During February 17–24, 2020, 345 samples, including 186 throat and 159 anal swabs, were collected from 195 *R. sinensis* in 10 different farms in Guangdong Province, China (Figure 1).

### 2.2. Sequence Reads Classification and Assembly

Using high-throughput sequencing results from the previous study (the accession number of the NCBI sequence archive is PRJNA751997), we redescribed the characteristics of the virome in *R. sinensis*. Briefly, high-quality clean reads were obtained using SOAPnuke software version 1.5.6 [22]. Ribosome and host sequences were removed using Burrows–Wheeler Alignment (BWA) software version 0.7.17 [23]. Then, clean reads were mapped to the virus reference data derived from the GenBank nonredundant nucleotide (NT) database by BWA software. Viral reads were preliminarily identified and counted at the level of the viral family. Reads of *Circoviridae* were further assembled by MEGAHIT version 1.1.2 [24], and contigs greater than 300 bp were compared to the GenBank nonredundant nucleotide and protein databases using BLASTn and BLASTx, respectively (the threshold of *E* value is 10^−5^).

### 2.3. Molecular Detection of Circoviruses in *Rhizomys sinensis*

Specific PCR was setup to determine the prevalence rate of the three identified circoviruses by viral metagenomic analysis. Primers were designed by Primer 5 software (Premier Biosoft International, Palo Alto, CA, USA) according to the contigs assembled above. In addition to the above three circoviruses, our PCR screening included rodent-associated circovirus 1–6. All primers are listed in Table S1.

Viral nucleic acid was extracted using RaPure Viral DNA/RNA Kit (Magen R4410-02, Guangzhou, China) according to the manufacturer's instructions, and target fragments were amplified with 2 × Taq Plus Master Mix Ⅱ (Vazyme P213, Nanjing, China). Purified PCR fragments by FastPure® Gel DNA Extraction Mini Kit (Vazyme DC301, Nanjing, China) were sequenced by the Tsingke Biotechnology (Guangzhou, China).

### 2.4. Genome Sequencing of Circoviruses in *Rhizomys sinensis*

Nine representative positive samples for circovirus were selected for whole-genome sequencing. In detail, the locations of contigs and the distance between contigs of the same virus were determined using the alignment results from MEGA 11. Then, the partial genome was amplified based on accurate genomic locations of contigs. Finally, based on the determined partial genomic sequences, the whole viral genome was amplified by using inverse PCR and genome walking. The purified genomic DNA using FastPure® Gel DNA Extraction Mini Kit (Vazyme DC301, Nanjing, China) was sequenced by the Tsingke Biotechnology (Guangzhou, China).

### 2.5. Sequence Comparison and Phylogenetic Analyses

The genomic sequences were assembled by SeqMan NGen®, version 7.1 (DNASTAR, Madison, WI) and aligned using MAFFT version 7.487 with the parameter L-INS-I [25]. Megalign (Lasergene, version 7.1) was used to determine the similarity of nucleotide and amino acid sequences, and MEGA 11 to determine the evolutionary relationships between strains of the present study and those from the GenBank database through phylogenetic analysis by the maximum likelihood (ML) method. ModelFinder Plus [26] was used to determine the most suitable evolution model for the phylogenetic tree of the whole and partial circoviral genome. The CLC sequence viewer software version 8.0 (QIAGEN Bioinformatics, Hilden, Germany) was used for the multiple sequence alignment of whole genome and PCV2d cap protein amino acid sequences.

Rep gene phylogenetic analysis included representative species of Circovirus genera. For the rodent-associated circovirus 7, all genomes and capsid protein (Cap) genes from the GenBank database were included as reference sequences. For PCV2, the phylogenetic analysis based on full genome and Cap genes included representative species of every subgenotype and other strains that infect nonpig species, especially rodents. Information on the reference genome retrieved from GenBank in this study is listed in Table S2.

### 2.6. Nucleotide Sequence Accession Numbers

All sequences obtained in the present study were submitted to GenBank under accession numbers OQ388322–OQ388330, and the previous metagenomic data have been deposited into the NCBI sequence archive under accession number PRJNA751997.

## 3. Results

### 3.1. Viral Metagenomic Overview

**Figure 2:**
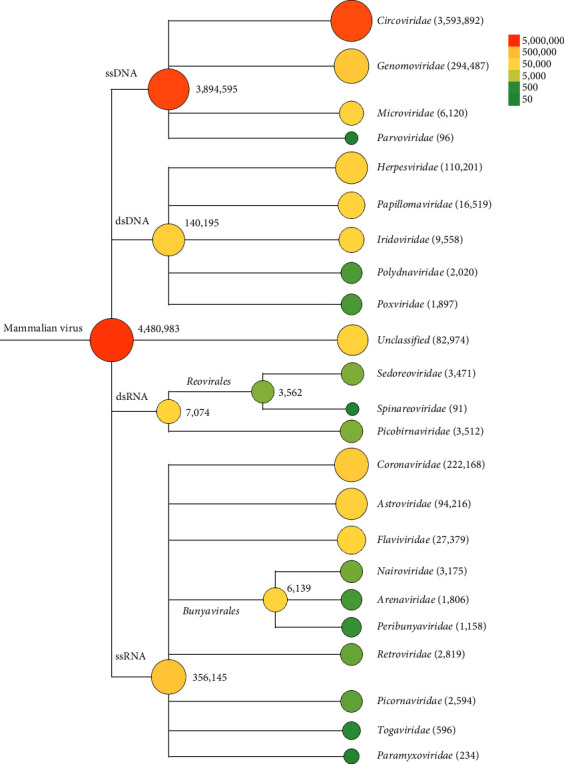
The classification of reads from bamboo rat. The diameters of circles next to the taxa are proportional to the logarithm of the total sequence reads. The color depth of the circles represents the number of reads, and the specific number of reads in different taxa is indicated in parentheses.

The throat and anal swabs analysis from *R. sinensis* revealed remarkable viral diversity. A total of 5,349,877 reads, which accounted for only 0.27% of the clean reads, had the best matches with viral genes or proteins using BLASTx and BLASTn. These viral reads were classified into 43 virus families, of which 4,480,983 matched 22 mammalian-related viral families, with DNA virus-related sequences being the most prevalent (90%) (Figure 2). Among mammalian DNA virus reads, viral sequences related to the *Circoviridae* family were the most abundant (89%) followed by *Genomoviridae* and *Parvoviridae*.

In light of the abundance of reads within the family *Circoviridae*, our attention was acutely directed toward the circoviruses. These samples from *R. sinensis* engendered an astonishing aggregation of 3,593,892 *Circoviridae*-related reads (Figure 2). These reads were distributed across seven distinct species: bamboo rat circovirus (3,590,165), rodent circovirus (607), porcine circovirus 2 (1,281), porcine circovirus-like virus (44), human fecal virus Jorvi2 (379), dromedary stool-associated circular ssDNA virus (85), and *Circoviridae* spp. (1,331). After merging the reads mentioned above, 36 contigs were assembled, with lengths ranging from 309 to 4,382 nucleotides (Table 1). The reclassification results showed that certain contigs initially identified as circoviruses may be erroneous sequences. This conclusion was reached because the contigs had limited nucleotide similarity with the reference sequence, and only a small portion could align with the reference sequence (Table 1).

### 3.2. Detection of Circoviruses in *Rhizomys sinensis*

In this study, we utilized specific primers to screen for the presence of bamboo rat circovirus, bamboo rat respiratory tract-associated circular ssDNA virus, and PCV2. We employed PCR to perform circoviruses tests on 345 samples (Figure 1 and Table 2). Among the *R. sinensis* of Guangdong Province, China, our results showed that the overall positive rates of bamboo rat circovirus, bamboo rat respiratory tract-associated circular ssDNA virus, and PCV2 were 64.1%, 44.1%, and 0.5%, respectively. Moreover, all regions were positive for bamboo rat circovirus and bamboo rat respiratory tract-associated circular ssDNA virus. Notably, over one-third of the 158 circovirus DNA-positive bamboo rats were coinfected with bamboo rat circovirus and bamboo rat respiratory tract-associated circular ssDNA virus (54/158). Conversely, PCV2 had an extremely low prevalence, and we detected its nucleic acid only in the throat swabs of one bamboo rat from Meizhou City, Guangdong Province. Furthermore, we also investigated the presence of rodent-associated circovirus 1–6, and our results demonstrated that all bamboo rats in this study were negative for these viruses.

### 3.3. Genetic Characterization of Bamboo Rat Circoviruses in *Rhizomys sinensis*

**Figure 3:**
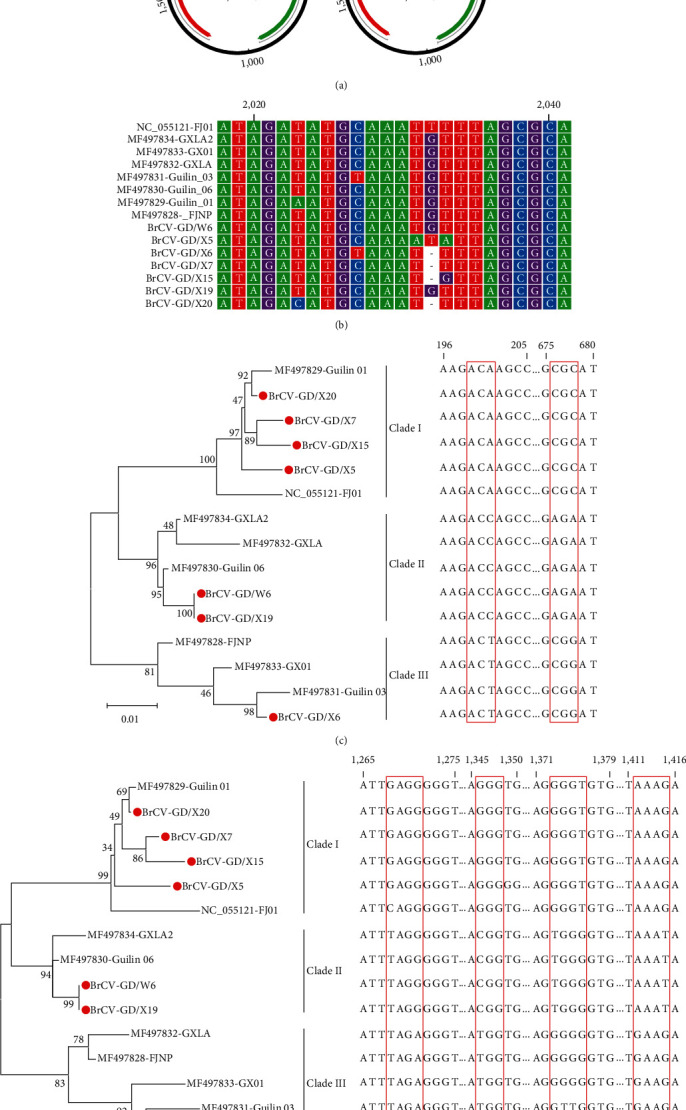
Identification of bamboo rat circoviruses in *Rhizomys sinensis*. (a) Genome schematic illustrating bamboo rat circoviruses' major open reading frames (ORFs) characteristics. Members of the species have two major ORFs and a conserved nonnucleotide motif marking the origin of replication. (b) Nucleotide sequence alignment of the partial region from all bamboo rat circoviruses was the only region in which single base deletions were present. (c and d) Phylogenetic analysis of the identified bamboo rat circoviruses by MEGA 11 software and the nucleotide sequence features observed by CLC sequence viewer software on the right side. The trees were constructed from the nucleotide sequences of the complete genome (c) and ORF2 (d). Bootstrap values expressed as percentages of 1,000 replications are shown at the branch nodes. The red circles represent bamboo rat circoviruses in this study. Nucleotide sequence features corresponding to each clade are displayed to the right of the phylogenetic tree, with regions marked by red boxes.

To investigate the genetic characteristics of prevalent bamboo rat circoviruses in South China, seven representative positive samples from different regions were utilized to obtain the complete genome sequences with a length of 2,111 or 2,112 nt (Figure 3(a)) and deposited in the GenBank database under accession number OQ388322-OQ388328. Notably, the ORF2 gene encoding the Cap protein had a length of either 906 or 657 nt, the latter due to a frameshift mutation caused by a one-base deletion at position 2,032 (Figure 3(b)). Pairwise sequence comparisons of the seven novel strains revealed a nucleotide sequence identity range of 95.8%–100% for the complete genome, 95.6%–100% for the Rep region, and 69.1%–100% for the Cap region. Furthermore, these strains showed a nucleotide sequence identity of 95.4%–99.5% for the complete genome, 95%–99.7% for the Rep region, and 68.2%–99.4% for the Cap region compared to the reference sequences obtained from GenBank data.

The results of the phylogenetic analysis showed that all bamboo rat circoviruses were divided into three clades (clades I, II, and III), and the seven Guangdong strains in our study were distributed in all three clades (Figures 3(c) and 3(d)). Remarkably, the alignment analysis of the complete genome and ORF2 gene sequences revealed unique nucleotide sequence characteristics in the three clades. Based on the complete genome, the phylogenetic tree indicated that clade I had ACA and CGC at positions 199–201 and 676–678, respectively. In contrast, clade II had ACC and AGA in these regions, while clade III had ACT and CGG (Figure 3(c)). In the ORF2 gene phylogenetic tree (Figure 3(d)), clade I was distinguished by GAGG, GGG, GGGT, and AAAG at positions 1,268–1,271, 1,346–1,348, 1,373–1,376, and 1,412–1,415, respectively. Conversely, clade II had TAGG, CGG, TGGG, and AAAT in these regions, and clade III had TAGA, TGG, GGGG/GTTG, and GAAG. These findings suggest distinctive nucleotide replacements across the three clades in these regions.

### 3.4. First Detection of Porcine Circovirus Type 2 in *Rhizomys sinensis*

We have identified a PCV2-like virus in *R. sinensis* samples using viral metagenomics analysis and specific PCR detection. Multiple sequence comparisons indicate that the *R. sinensis* origin (RS origin) PCV2 strain shares 94.9%–99.5%, 96.8%–99.8%, and 88.9%–99.3% nucleotide similarity with previously reported PCV2 sequences in the complete genome, ORF1, and ORF2, respectively, from diverse species in the GenBank database. Based on the guidelines from the ICTV report [1], this isolate was classified as PCV2 and designated RtRs-PCV2/2020. To our knowledge, this represents the first report of a PCV2 strain detected from *R. sinensis*. We have obtained the complete genome sequence of this RS-origin PCV2 strain, which spans 1,767 nucleotides. The sequence has been deposited in the GenBank database under accession number OQ388329. Notably, the RS-origin PCV2 strain exhibits 95%–97.6% (complete genome), 98.1%–99.8% (Rep), and 89.9%–94.4% (Cap) nucleotide sequence identity with other rodent-origin PCV2 strains. Moreover, it shows 97.5%–98.9% (complete genome), 99.0%–99.6% (Rep), and 94.4%–98.9% (Cap) nucleotide sequence identity with porcine-origin PCV2 strains from Meizhou City.

**Figure 4:**
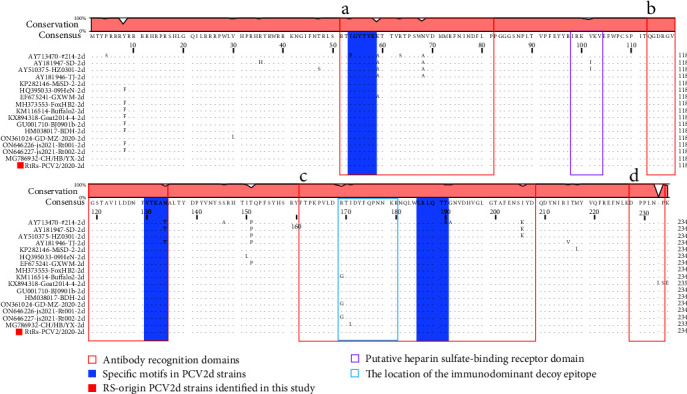
Multiple sequence alignment of Cap protein amino acid sequences from PCV2d strains of diverse origins. The alignment includes the RS-origin PCV2 strain (RtRs-PCV2/2020) and the other 16 reference PCV2d strains. The blue areas denote the unique motifs in PCV2d Cap protein sequences. Antibody recognition domains, heparin sulfate-binding receptor domain, and immunodominant decoy epitope are indicated by red, purple, and blue boxes, respectively. The red squares indicate the strain of this study.

Compared to the amino acid sequences of PCV2d strains from different origins (*n* = 16), the ORF2-encoded 234 amino acids of the RS-origin PCV2 strain were relatively conserved without any specific substitution (Figure 4). In this study, the RS-origin PCV2d strain also possessed the typical motifs for PCV2d, and four antigenic domains (designated as epitopes a–d), one immunodominant bait epitope within epitope c, and one heparin-binding motif were the same as the PCV2d strains from other species. In this study, the RS-origin PCV2 strain also possessed the typical motifs ^53^IGYTVK^58^, ^130^VTKAN^134^, and ^185^LRLQTT^190^ for PCV2d. Moreover, four antigenic domains (designated as epitopes a–d), one immunodominant bait epitope within epitope c, and one heparin-binding motif were identified in the predicted amino acid sequence of the Cap protein of the RS-origin PCV2 strain (Figure 4). Furthermore, the same critical amino acid residues were found in the four antigenic domains, including D-70, M-71, N-77, and D-78 in epitope a, Q-113, D-115, and D-127 in epitope b, Y-173, F-174, Q-175, and K-179 in epitope c, as well as E-203, I-206, and Y-207 in epitope d (Figure 4).

**Figure 5:**
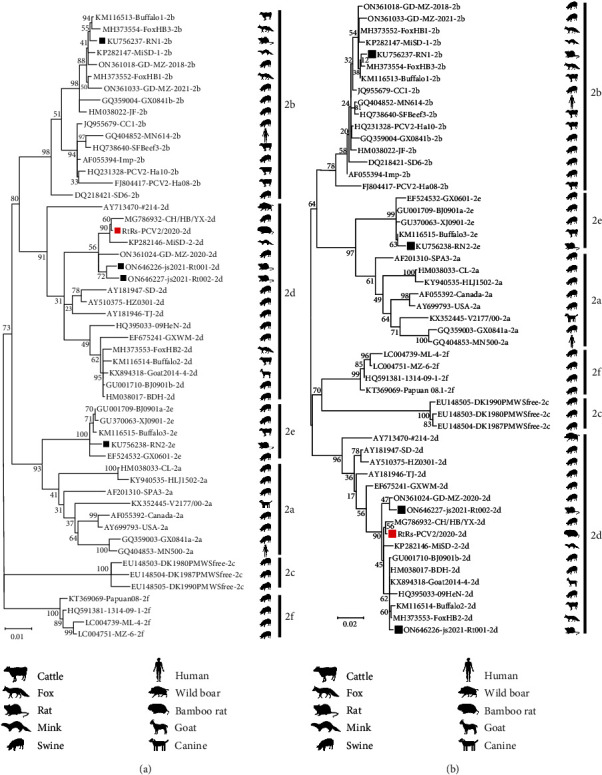
Phylogenetic analysis of our RS-origin PCV2 strain and reference PCV2 strains. (a) Phylogenetic tree based on PCV2 complete genome sequences; (b) phylogenetic tree based on PCV2 Cap gene sequences. Both phylogenetic trees were constructed by the ML method with the TN93 model using MEGA 11 software. One thousand bootstrap replications were used. The RS-origin and other rodent-origin PCV2 strains were indicated by red and black squares, respectively. The host origin of each strain was marked to the right of the strain with the corresponding species shadow.

To determine the genetic relationships of the RS-origin PCV2 strain compared to other representative PCV2 strains, two phylogenetic trees based on the complete genome and ORF2 gene were constructed separately. Our phylogenetic analysis revealed that RtRs-PCV2/2020 belongs to the PCV2d genotype (Figure 5), first identified in *R. sinensis*. The RS-origin PCV2 strain and the porcine-origin PCV2 strain (GD-MZ-2020) clustered in the same subclade (Figure 5).

### 3.5. A Novel Rodent-Associated Circovirus Was Identified in *Rhizomys sinensis*

A 593-nucleotide contig was obtained from *R. sinensis* by Illumina sequencing, which was identified as a member of the *Circoviridae* family (Table 1). This contig matched the ORF1 gene (Rep) of dromedary stool-associated circular ssDNA virus isolate DcSCV_c1000 (KM573764) (Figure S1(a)), and there was 74.87% nucleotide identity between them. Although efforts were made to obtain the complete genome sequence through inverse PCR and gene-walking strategies, whole genome amplification failed due to low viral copy numbers and limited fluid availability in the collected swab samples. Phylogenetic analysis of the partial putative replicase protein gene revealed that the virus belonged to a distinct clade within the *Circoviridae* family, along with camel-origin circovirus DcSCV_c1000, two BatACV-13 strains, and whale-origin circovirus IP13001 (Figure S1(b)). In summary, we speculate that this virus represents a novel species within the family *Circoviridae* and is named bamboo rat-associated circular ssDNA virus isolate BrRCV-GD/X15.

## 4. Discussion

To identify all viruses through sequence similarity searches, viral metagenomics provides an opportunity to comprehensively investigate the viral community composition of a particular host species or environment [27, 28]. Over the past two decades, it has successfully identified the etiological agents of emerging or reemerging infectious disease outbreaks in humans and animals [10, 20, 29, 30]. Previous studies have characterized the viral flora of several species of rodents and uncovered a significant diversity of novel viruses [10, 20, 31], highlighting the importance of these animals as reservoirs of zoonotic pathogens such as hantavirus [32], arenavirus [17, 33], hepatitis E viruses, and coronaviruses [10]. Therefore, the risk of emerging infectious diseases in rodents should be considered.

In this study, we investigated the features of the vertebrate-associated viral community in pharyngeal and anal swabs from *R. sinensis* in Guangdong Province. Our findings showed that the identified viral sequences were closely or distantly related to known viruses. Surprisingly, we detected a high abundance of DNA viral sequences in *R. sinensis*, especially those belonging to the family *Circoviridae*, which differed significantly from the results reported by He et al. [20]. These differences may be attributed to variations in the sampling techniques, sample types, health status of the animals, and/or research methodologies between the two studies. Additionally, the findings of this study align with prior research suggesting the prevalence of plant and insect viruses in rodents, emphasizing the importance of dietary patterns when considering the viral communities associated with these animals [31].

Recently, many Rep-containing circular DNA genomes have been discovered in diverse animals and environments, considerably expanding the genetic diversity within the *Circoviridae* family. Consequently, we became interested in exploring the genetic diversity of circoviruses carried by *R. sinensis* after identifying numerous circovirus nucleotide sequences within this species using viral metagenomics.

Our results indicated that bamboo rat circovirus is widely prevalent in *R. sinensis* in Guangdong, and seven complete genome sequences of bamboo rat circovirus were identified, consisting of two lengths, 2,111 and 2,112 nt, with the shorter sequence exhibiting a frameshift mutation in the ORF2 gene due to a single base deletion. These strains shared high nucleotide identity with reference sequences at the whole genome level. Phylogenetic analysis revealed that bamboo rat circoviruses were grouped into three main clades, and those strains identified in this study were distributed across all three clades, with clade I being dominant. Interestingly, specific nucleotide substitutions were found at multiple locations in the viral genome, corresponding to different clades of bamboo rat circovirus. We hypothesize that these sites are essential for the evolutionary clustering of bamboo rat circovirus, but this hypothesis requires further confirmation with more extensive data.

Our study identified PCV2 in *R. sinensis*, which represents the first report of PCV2 in bamboo rats. Our findings confirm the presence of PCV2 in rodents and provide evidence of cross-species transmission of PCV2 from swine to bamboo rats, consistent with previous reports of PCV2 circulation in rodents [34, 35]. Furthermore, our multiple sequence alignment and phylogenetic analysis indicate that the RS-origin PCV2 strain shares high similarity with porcine-origin PCV2 strains, particularly those prevalent in local swine herds, consistent with previous reports [34, 36]. Our phylogenetic analysis revealed that RS-origin PCV2 belongs to the PCV2d genotype, distinct from the PCV2b and PCV2e genotypes previously identified in rodents [36]. The finding is consistent with a recent report by Zhao et al. [37] who detected PCV2d in wild rats collected in 2021, further supporting the ability of PCV2d to cross the species barrier between pigs and rodents. Our results suggest that the strain of PCV2d from rodents in China can be traced back to at least 2020. However, the exact time when PCV2d acquired the capacity for cross-species transmission between pigs and rodents remains to be discovered, even though the earliest detection of PCV2d in swine herds dates back to 2002 [38]. Therefore, retrospective studies on rodent samples are necessary to identify the temporal node at which PCV2d acquired the capacity for interspecies transmission. These findings will expand our understanding of the host range of PCV2 and provide insight into the genetic evolution and epidemiology of rodent-origin PCV2, which is essential for disease control and prevention.

In addition, through metagenomic sequencing on bamboo rat samples, we identified a circovirus sequence with the highest nucleotide identity (74.87%) to the partial Rep gene of the dromedary stool-associated circular ssDNA virus. Based on the guidelines from the ICTV report [1], we hypothesized this to be a novel circovirus and named it bamboo rat-associated circular ssDNA virus, which was supported by phylogenetic analysis based on the partial putative Rep protein gene (ORF1). This virus is distinct from rodent-associated circovirus 1–7 obtained in a previous study [10]. It appears closely related to circoviruses from dromedaries, bats, and whales. Despite our efforts to obtain the complete genomic data of GD/BrRCV-X15 detected in *R. sinensis*, we were unsuccessful, which restricted the analysis of the genetic diversity of circoviruses. We could not obtain additional samples for further analysis due to the ban on farming wild animals in China since February 24, 2020. Therefore, while the data we have collected are valuable, caution should be exercised in their interpretation.

## 5. Conclusions

In summary, this study characterized the virome of *R. sinensis* in Guangdong Province and identified the genetic diversity of circoviruses in this species for the first time. Bamboo rat circovirus was the most prevalent, and all strains exhibited specific nucleotide substitution patterns, which allowed us to divide them into three distinct evolutionary groups. For the first time, we also report the presence of the PCV2d genotype in *R. sinensis*, which enriches our knowledge of the genetic diversity and host range of PCV2. At the same time, the Cap proteins of RS-origin and porcine-origin PCV2d share common antibody recognition domains, immunodominant decoy epitope regions, and heparin sulfate-binding motifs, indicating conserved structural features of this viral protein among these strains. Finally, we identified a novel circovirus species in *R. sinensis*, closely related to circoviruses from camels, bats, and whales. Overall, for the first time, the findings of this study provide important insights into the pathogen background of *R. sinensis* and shed new light on the diversity, evolution, and interspecies transmission of circoviruses, highlighting the significance of continued surveillance of viruses in wildlife populations.

## Figures and Tables

**Table 1:** The information of the contigs assembled from circovirus reads in this study.

Reference virus	Number of matched contig	Contigs length (nt)	Nucleotide identity (%)	Query cover (%)	Geographical location
Bamboo rat circovirus	17	357–2,239	68.32–100	9–100	Shaoguan, Zhanjiang, Yunfu
Porcine circovirus 2	2	496, 1,105	97.30–99.8	100	Meizhou
Dromedary stool-associated circular ssDNA virus	1	593	74.87	100	Zhanjiang
Rodent circovirus	7	448–3,615	68–94.74	1–33	Shaoguan, Zhanjiang, Yangjiang
Porcine circovirus-like virus	2	472, 476	70.3–88.71	7–11	Shaoguan
Human fecal virus Jorvi2	1	3,628	74.05	3	Shaoguan
*Circoviridae* spp.	6	309–4,382	64.19–90.57	1–30	Shaoguan, Zhanjiang

**Table 2:** Results of detection of multiple circoviruses nucleic acids in different cities in Guangdong.

City	Region	No. positive/no. of *Rhizomys sinensis* (%)		
		Bamboo rat circovirus	Bamboo rat-associated circular ssDNA virus	PCV2
Zhanjiang	W01	16/20 (80)	5/20 (25)	0/20 (0)
	W03	20/20 (100)	2/20 (10)	0/20 (0)
Yangjiang	W07	18/20 (90)	2/20 (10)	0/20 (0)
Yunfu	W09	9/21 (42.9)	13/21 (61.9)	0/21 (0)
	W10	18/21 (85.7)	13/21 (61.9)	0/21 (0)
Guangzhou	Z03	17/21 (80.9)	13/21 (61.9)	0/21 (0)
Shaoguan	N01	17/20 (85)	11/20 (55)	0/20 (0)
	N05	4/20 (20)	15/20 (75)	0/20 (0)
Heyuan	E10	5/15 (33.3)	5/15 (33.3)	0/15 (0)
Meizhou	E02	1/17 (5.9)	7/17 (41.2)	1/17 (5.9)
Total		125/195 (64.1)	86/195 (44.1)	1/195 (0.5)

## Data Availability

The data supporting this study's findings are available in the article and the supplementary material.

## Supplementary Materials

Supplementary Materials Table S1: the information on the detection primers for circoviruses in *Rhizomys sinensis* in this study. Table S2: information of reference sequences used in this study. Figure S1: identification of a novel species of the family Circoviridae in *Rhizomys sinensis*.
