# Supplementary material for: The Genetic Diversity and Interspecific Transmission of Circovirus in Rhizomys sinensis in Guangdong, Southern China
Source: Transbound Emerg Dis. 2023 Nov 9;2023:6668569. doi: 10.1155/2023/6668569 (PMC12017068; doi:10.1155/2023/6668569)

**Supplemental material**

The genetic diversity and interspecific transmission of circovirus in *Rhizomys sinensis* in Guangdong, southern China

Zhaowen Ren^a,b^†‡, Zi-Guo Yuan^b^†, Shengjun Luo^a^†, Chenglong Sun^c^, Pian zhang^a^, Jieshi Yu^d^, Xiaofan Chen^a,b^, Jinpin Chen^e^⸸, Yan Hua^f^, Gang Wang^a^, Hua Xiang^a^, Rujian Cai^a^, Jing Chen^a^, Yuan Huang^a^, Hao Yuan^b^, Na Li^a,b^, Ming Liao^a,b^*, Xiaohu Wang^a^*

**Supplementary Tables**

**Table S1.** **The information on the detection primers for Circoviruses in *Rhizomys sinensis* in this study.**

| **Virus name** | **Primer name** | **Primer sequences (5’ to 3’)** | **Product length (nt)** |
| --- | --- | --- | --- |
| Rodent-associated circovirus 1 | RCV-1-F | GAGGAGGAGCAAGCAGTGAT | 327 |
|  | RCV-1-R | ACGAGCAACATGGCGGATA |  |
| Rodent-associated circovirus 2 | RCV-2-F | CATGAGGAAGATGCTGTGAAGG | 685 |
|  | RCV-2-R | GGTTCTTGTTGCTGGTGATGA |  |
| Rodent-associated circovirus 3 | RCV-3-F | CCTGCTCGTGGTGATGATTG | 557 |
|  | RCV-3-R | GGCGTCCTTGTTGGTAATCTC |  |
| Rodent-associated circovirus 4 | RCV-4-Rep-F | ATGGCACAGGCTCAGGTTC | 285 |
|  | RCV-4-Rep-R | GCGATTGGCGGTATCATCTC |  |
| Rodent-associated circovirus 5 | RCV-5-F | CGGAGCAAGCCTCTGAGTAT | 262 |
|  | RCV-5-R | CCAGGTGGACCAATCAATACAAT |  |
| Rodent-associated circovirus 6 | RCV-6-Rep-F | CGAAGGTGGCGACTGACATA | 209 |
|  | RCV-6-Rep-R | TAGACGAATCATCCGACGAAGA |  |
| Rodent-associated circovirus 7 (bamboo rat circovirus) | Rs-CriF | GGTGTCCAGTCTCATAGTCA | 234 |
|  | Rs-CriR | CACCAAAGCCTCAGAGAACT |  |
| Bamboo rat-associated circular ssDNA virus isolate BrRCV-GD/X15 | GD/BrRCV-F | CTCCTCAACAGTGCGCTAAT | 263 |
|  | GD/BrRCV-R | GGTGCACATGTTTCTTCTGA |  |
| Porcine circovirus type 2 | PCV2all_F | GGGTCTTTHAKATTAAATTC | 680 |
|  | PCV2all_R | ATGACGTATCCAAGGAG |  |

**Table S2. Information of reference sequences used in this study.**

| **Strain name** | **Organism** | **Source** | **GenBank Accession no.** |
| --- | --- | --- | --- |
| MN614 | PCV2 | Homo | GQ404852 |
| SFBeef | PCV2 | Beef | HQ738640 |
| Ha10 | PCV2 | calf | HQ231328 |
| CC1 | PCV2 | swine | JQ955679 |
| Imp | PCV2 | swine | AF055394 |
| Ha08 | PCV2 | calf | FJ804417 |
| GX0841b | PCV2 | swine | GQ359004 |
| JF | PCV2 | swine | HM038022 |
| FoxHB1 | PCV2 | fox | MH373552 |
| MiSD-1 | PCV2 | Mink | KP282147 |
| Buffalo1 | PCV2 | Beef | KM116513 |
| FoxHB3 | PCV2 | fox | MH373554 |
| SD6 | PCV2 | swine | DQ218421 |
| #214 | PCV2 | wild boar | AY713470 |
| SD | PCV2 | swine | AY181947 |
| HZ0301 | PCV2 | swine | AY510375 |
| TJ | PCV2 | swine | AY181946 |
| MiSD-2 | PCV2 | Mink | KP282146 |
| 09HeN | PCV2 | swine | HQ395033 |
| GXWM | PCV2 | swine | EF675241 |
| FoxHB2 | PCV2 | fox | MH373553 |
| Buffalo2 | PCV2 | Beef | KM116514 |
| Goat2014-4 | PCV2 | goat | KX894318 |
| BJ0901b | PCV2 | swine | GU001710 |
| BDH | PCV2 | swine | HM038017 |
| BJ0901a | PCV2 | swine | GU001709 |
| XJ0901 | PCV2 | swine | GU370063 |
| Buffalo3 | PCV2 | Beef | KM116515 |
| GX0601 | PCV2 | swine | EF524532 |
| CL | PCV2 | swine | HM038033 |
| HLJ1502 | PCV2 | swine | KY940535 |
| SPA3 | PCV2 | swine | AF201310 |
| V2177/00 | PCV2 | dog | KX352445 |
| Canada | PCV2 | swine | AF055392 |
| USA | PCV2 | swine | AY699793 |
| GX0841a | PCV2 | swine | GQ359003 |
| MN500 | PCV2 | Homo | GQ404853 |
| Papuan 08.1 | PCV2 | swine | KT369069 |
| 1314-09-1 | PCV2 | swine | HQ591381 |
| ML-4 | PCV2 | swine | LC004739 |
| MZ-6 | PCV2 | swine | LC004751 |
| DK1990PMWSfree | PCV2 | swine | EU148505 |
| DK1980PMWSfree | PCV2 | swine | EU148503 |
| DK1987PMWSfree | PCV2 | swine | EU148504 |
| GD-MZ-2018 | PCV2 | swine | ON361018 |
| GD-MZ-2020 | PCV2 | swine | ON361024 |
| GD-MZ-2021 | PCV2 | swine | ON361033 |
| RN2 | PCV2 | Rat | KU756238 |
| RN1 | PCV2 | Rat | KU756237 |
| CH/HB/YX | PCV2 | swine | MG786932 |
| js2021-Rt001 | PCV2 | Rat | ON646226 |
| js2021-Rt002 | PCV2 | Rat | ON646227 |
| FJ01 | BRCV | Bamboo rat | NC_055121 |
| GXLA2 | BRCV | Bamboo rat | MF497834 |
| GX01 | BRCV | Bamboo rat | MF497833 |
| GXLA | BRCV | Bamboo rat | MF497832 |
| Guilin 03 | BRCV | Bamboo rat | MF497831 |
| Guilin 06 | BRCV | Bamboo rat | MF497830 |
| Guilin 01 | BRCV | Bamboo rat | MF497829 |
| FJNP | BRCV | Bamboo rat | MF497828 |
| BaCV1 | BarCV | Barbel | NC_015399 |
| XOR | BatACV-1 | Bat | NC_038385 |
| XOR7 | BatACV-2 | Bat | NC_021206 |
| RfCV-1 | BatACV-3 | Bat | NC_038386 |
| TbCV-1 | BatACV-4 | Bat | NC_028045 |
| BtPa-CV-1/NX2013 | BatACV-5 | Bat | NC_038387 |
| BtRa-CV/JS2013 | BatACV-6 | Bat | NC_038388 |
| BtRs-CV/HuB2013 | BatACV-7 | Bat | NC_038389 |
| BtMr-CV/GD2012 | BatACV-8 | Bat | NC_038390 |
| BtRf-CV-61/YN2010 | BatACV-9 | Bat | NC_039033 |
| HK00220 | BatACV-10 | Bat | LC456718 |
| Mengyuan2 | BatACV-11 | Bat | NC_055122 |
| B18-157 | BatACV-12 | Bat | MW732037 |
| BatACV/Mm1/Switzerland/2019 | BatACV-13 | Bat | MT815980 |
| BatACV/Rh1/Switzerland/2019 | BatACV-13 | Bat | MT815981 |
| BatACV/BtVm/Switzerland/2019 | BatACV-13 | Bat | MT815982 |
| Beak and feather disease virus | BFDV | Parrot | NC_001944 |
| UaCV/Reno/2014 | Bear circovirus | Bear | MN371255 |
| Canary circovirus | CCV | Canary | NC_003410 |
| UCD1-1698 | CanineCV | Canine | NC_020904 |
| Chimp17 | ChimpACV-1 | Chimpanzee | NC_038391 |
| Pl-CV3 | Pl-CV | palm civet | NC_040679 |
| 33753-52 | DuCV | Duck | NC_007220 |
| Banff/2019 | ElkCV | Cervus canadensis | MN585201 |
| H5 | EcatfishCV | European catfish | NC_025246 |
| Finch circovirus | FiCV | finch | NC_008522 |
| GCV | GoCV | Goose | NC_003054 |
| Gull circovirus | GuCV | gull | NC_008521 |
| NG13 | HuACV-1 | Homo | NC_038392 |
| MiCV-DL13 | MiCV | mink | NC_023885 |
| CCirVL/Fresno | CCirVL | mosquito | NC_040833 |
| Croz_chick | PeCV | Penguin | NC_055590 |
| CoCV | PiCV | pigeon | NC_002361 |
| PCV | PCV1 | swine | NC_001792 |
| pmws PCV | PCV2 | swine | AF027217 |
| 29160 | PCV3 | swine | NC_031753 |
| HNU-AHG1-2019 | PCV4 | swine | NC_055580 |
| 4-1131 | RaCV | Corvus coronoides | NC_008375 |
| RtMc-CV-1/Tibet2014 | RodentACV-1 | Rodent | NC_055152 |
| RtAc-CV-2/GZ2015 | RodentACV-2 | Rodent | NC_055155 |
| RtMc-CV-2/Tibet2014 | RodentACV-3 | Rodent | NC_055154 |
| RtAs-CV/IM2014 | RodentACV-4 | Rodent | NC_055151 |
| RtNe-CV/YN2013 | RodentACV-5 | Rodent | NC_055149 |
| RtAd-CV/SAX2015 | RodentACV-6 | Rodent | NC_055153 |
| FJ01 | RodentACV-7 | Bamboo rat | NC_055121 |
| Starling circovirus | StCV | European Starling | NC_008033 |
| H51 | SwCV | mute swan | NC_025247 |
| A1 | TickACV-1 | tick | NC_030199 |
| hlj-Ic.518 | TickACV-2 | tick | NC_055123 |
| IP13001 | WCV | whale | MN103538 |
| 8454V25-1 | ZfiCV | Zebra Finch | NC_026945 |
| DcSCV_c1000 | DcSCV | dromedary camel | KM573764 |

**Supplementary Figures**

**Figure S1.** Identification of a novel species of the family Circoviridae in Rhizomys sinensis. (A) The genomic location of Bamboo rat associated circular ssDNA virus isolate GD/BrRCV-X15 compared with reference strain DcSCV_c1000. (B) Phylogenetic tree based on partial ORF1 sequences. The ML method constructed the phylogenetic tree with the GTR model using MEGA 11 software. One thousand bootstrap replications were used. The Bamboo rat-associated circular ssDNA virus isolate BrRCV-GD/X15 obtained in this study was indicated by a red triangle.


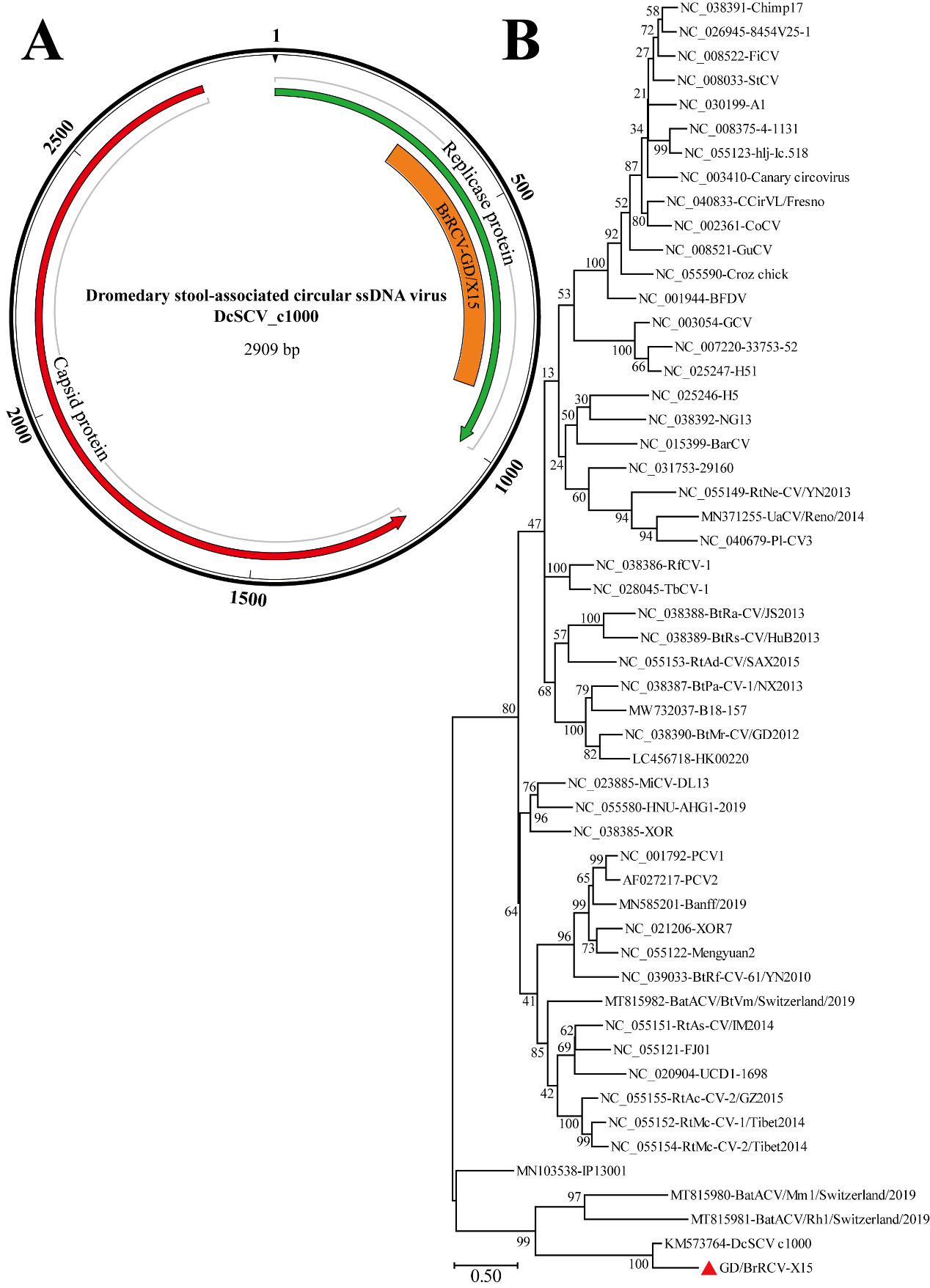

Supplement: Supplementary Materials — Table S1: the information on the detection primers for circoviruses in Rhizomys sinensis in this study. Table S2: information of reference sequences used in this study. Figure S1: identification of a novel species of the family Circoviridae in Rhizomys sinensis. [file 6668569.f1.docx]
